# Psychometric Validation of the Living with Chronic Illness Scale in Patients with Chronic Heart Failure

**DOI:** 10.3390/ijerph18020572

**Published:** 2021-01-12

**Authors:** Leire Ambrosio, David Perez-Manchon, Gloria Carvajal-Carrascal, Alejandra Fuentes-Ramirez, Neus Caparros, Manuel Ignacio Ruiz de Ocenda, Eva Timonet, Maria Victoria Navarta-Sanchez, Carmen Rodriguez-Blazquez

**Affiliations:** 1 School of Health Sciences, University of Southampton, Southampton SO17 1BJ, UK; 2 Faculty of Health, Camilo Jose Cela University, 28692 Madrid, Spain; dperezman@gmail.com; 3 Facultad de Enfermería y Rehabilitación, Universidad de La Sabana, Bogotá 53753, Colombia; gloria.carvajal@unisabana.edu.co (G.C.-C.); alejandra.fuentes@unisabana.edu.co (A.F.-R.); 4 Faculty of Legal and Social Science, La Rioja University, 26004 La Rioja, Spain; caparros@unirioja.es; 5 La Rioja Health Care System, 26001 La Rioja, Spain; mrocenda@riojasalud.es; 6 Department of Cardiology, Costa del Sol Hospital, 29603 Malaga, Spain; evamta@hcs.es; 7 Faculty of Medicine, Autonomous University of Madrid, 28029 Madrid, Spain; maria.navarta@uam.es; 8 National Centre of Epidemiology and CIBERNED, Carlos III Institute of Health, 28029 Madrid, Spain; crodb@isciii.es

**Keywords:** chronic illness, chronic heart failure, psychometric properties, self-reported outcome, instrument

## Abstract

It is necessary to develop self-reported instruments that evaluate the process of living with chronic heart failure (HF) holistically. The Living with Chronic Illness Scale—HF (LW-CI-HF) is the only available tool to evaluate how patients are living with HF. The aim is to analyse the psychometric properties of the LW-CI scale in the HF population. An international, cross-sectional validation study was carried out in 603 patients living with HF from Spain and Colombia. The variables measured were living with HF, perceived social support, satisfaction with life, quality of life and global impression of severity. The LW-CI-HF scale presented good data quality and acceptability. All domains showed high internal consistency with Cronbach’s alpha coefficient ≥ 0.7. The intraclass correlation coefficient for the total score was satisfactory (0.9) in test–retest reliability. The LW-CI-HF correlated 0.7 with social support and quality of life measures. Standard error of measurement was 6.5 for total scale. The LW-CI-HF scale is feasible, reliable and valid. However, results should be taken with caution in order to be used in clinical practice to evaluate the complex process of living with HF. Further research is proposed.

## 1. Introduction

Nowadays, cardiovascular diseases are a major health problem and the number one cause of death worldwide [1]. Chronic heart failure (HF) is a clinical chronic disease resulting in reduced cardiac activity. Its prevalence has increased in recent years, particularly in people over 70 years of age [2]. Approximately 26 million people worldwide live with HF, and the prevalence is expected to increase in the coming years [3]. People living with HF have symptoms typically including dyspnoea, oedema, pain, depression, fatigue, sleep disturbance or anxiety and signs caused by a structural cardiac abnormality [4]. HF is a chronic disease that causes great physical and social problems [5] and is characterized by a progressive deterioration in the quality of life, marked by stages of severity of symptoms and exercise intolerance [6]. HF patients have more unpredictable and less sequential stages than those with other chronic illnesses, such as cancer, chronic obstructive pulmonary disease, acquired immune deficiency syndrome or diabetes mellitus [7,8].

When living with HF, patients must change their lifestyles and acquire self-management habits while coping to manage day-to-day challenges [9,10]. Living with HF is a multidimensional chronic process, including internal processes such as acceptance and coping with the disease, self-management of the symptoms and treatment, as well as integration and adjustment to the new life situation that HF generates [11,12]. Accepting HF implies the absence of feelings of denial or anger, where the person acknowledges and assumes the fact that they have HF [11,12]. Coping refers to the process of learning to face HF and implementing different strategies to deal with the disease [9,10,11]. Self-management refers to having some knowledge about the HF itself, adhering to a plan and actively participating in the decision-making that the illness involves [11,12]. Finally, integrating the HF implies making changes in lifestyle to search for a new normal and adjusting entails a progressive process of the transformation of the person’s self-identity, as the HF also forms part of it [9,10,11,12]. Clinical specialists and, more specifically, cardiovascular nurses have a key role in empowering patients to make autonomous decisions in relation to the process of living with HF because they can facilitate the process and consequently help improve satisfaction, buffer stress and promote adherence and a healthy lifestyle in patients [12,13]. To achieve this, having instruments to identify and evaluate the impact of HF clinical condition on patients’ daily lives would be a great facilitator to identify key aspects and design an individualized care plan according to the patient’s needs.

To our knowledge, the Living with Chronic Illness (LW-CI) (in Spanish: *Escala de convivenvia con un proceso crónico EC-PC*) Scale is the only instrument available in clinical practice and research that evaluates the process of living with one or more chronic conditions in a personalised way, focusing on the person and not on the disease [14]. The LW-CI scale has been piloted in Spain and South America in some prototypical and prevalent chronic conditions, such as Parkinson’s disease [15], type 2 diabetes mellitus and chronic obstructive pulmonary disease [16], with satisfactory preliminary psychometric results. However, no studies have been carried out to determinate whether the LW-CI scale is reliable and valid for use in patients living with HF in a Spanish-speaking population. In this sense, testing the LW-CI scale in patients with HF (LW-CI-HF scale) will provide an innovative measure that could permit the identification of the factors that helps a person to live positively with the illness and consequently, design individualized care interventions that address the actual needs of each patient.

The aim of this study is to analyse the psychometric properties of the LW-CI-HF scale in the Spanish-speaking population living with HF.

## 2. Materials and Methods

### 2.1. Design

An observational and cross-sectional study with a retest was carried out in different healthcare from Spain and Colombia.

This study is part of a bigger and ambitious research project with the general aim to achieve a standardized and unique self-reported scale in Spanish-speaking population to evaluate the process of living with one or more than one chronic diseases, such as diabetes mellitus type 2, rheumatoid and osteoarthritis, chronic heart failure or Parkinson’s disease, among others. The present study is part of a series of validation studies carried out in different chronic diseases and countries [15,16], focusing on the validation of the scale in patients living with HF in Spain and Colombia.

### 2.2. Sample, Sampling and Sample Size

We applied a consecutive case sampling [17,18] to participant identification in the primary and secondary healthcare systems from Spain and Colombia. The sample was composed of people living with HF who met the following inclusion criteria: (a) having been diagnosed with HF by a general practitioner or a cardiologist; (b) any duration of HF; (c) adult people older than 18 years old; (d) being able to read, understand and answer written questionnaires; (e) living in the community and not being hospitalised at the time of the study; (f) being a Spanish or Colombian native; (g) written documentation of informed consent. Exclusion criteria were (a) having been diagnosed with cognitive deterioration and/or current psychiatric disorders by a clinical practitioner (e.g., general practitioner or psychiatrist) and (b) neurological comorbidities or any other disorder that could interfere with or impede the study, such as answering self-reported scales. According to international criteria [19] a sample size of at least 10 patients per item of the validated scale (LW-CI-HF scale) was established. In this way, for this validation study, a minimum sample size of 260 patients living with HF was sought, as the LW-CI-HF scale is a 26-item measure.

### 2.3. Patient and Public Involvement

Patient and public involvement was examined during the pilot testing to the scale, before starting the official data collection for this validation study. As shown in the previous study carried out to this validation study, patients and caregivers were directly involved in the design and pilot phase through qualitative and quantitative methodologies [16]. Patient and public involvement was key to achieve the final version of the LW-CI-HF scale to be validated. For this validation study, an ad hoc questionnaire related to the LW-CI-HF scale was completed to analyse its adequacy and content validity. Patients’ opinions on the relevance and usefulness of the scale in clinical practice and for their daily living, or its length, were collected through the ad hoc questionnaire.

### 2.4. Measuring Scales

In addition to the Spanish version of the LW-CI-HF scale, patient-reported measures and the Spanish validated versions of the following self-reported measures were used for this validation study, as follows:

#### 2.4.1. Sociodemographic Questionnaire and Historical Questionnaire

We included a short sociodemographic questionnaire and historical data for HF to collect information related to the patient and the disease. We included variables related to the person, such as age, gender, marital status, employment situation and educational level. Besides, we included variables related to the disease as age at diagnosis, duration of HF and severity of their symptoms according to functional classification using the New York Heart Association (NYHA) recommendations [13].

#### 2.4.2. Living with Chronic Illness—Chronic Heart Failure Scale

The LW-CI-HF scale is a self-reported measuring scale to evaluate the complex process of living with HF. It was developed based on empirical and conceptual studies carried out by international experts in the field of long-term conditions [11,20]. The LW-CI-HF scale is a 26-item scale grouped into five domains: Domain 1—Acceptance (4 items); Domain 2—Coping (7 items); Domain 3—Self-management (4 items); Domain 4—Integration (5 items); Domain 5—Adjustment (6 items) [11]. All items are answered using a 5-point Likert scale from never or nothing (0) to always or a lot (4), except for Domain 1—Acceptance, which is reversely scored from never or nothing (4) to always or a lot (0). The LW-CI-HF scale has total score value from 0 points, indicating negative living with HF, to 104 points, reflecting positive living with HF [16]. The results from previous studies [15,16] showed satisfactory psychometric properties in patients with other chronic conditions such as Parkinson’s disease (Cronbach’s alpha values ranged between 0.7 and 0.9 and internal validity correlations ranged from 0.5 to 0.8) [15].

#### 2.4.3. Duke–UNC Functional Social Support Questionnaire (DUFSS)

The DUFSS was used in this validation study to evaluate functional social support [21,22]. The DUFSS was measured using 11 items containing areas defined as confidant, affective and instrumental support. Each item is scored on a 1 to 5 scale. “Much less than I would like” receives a score of 1, and “as much as I would like” receives a score of 5. The total score ranged from 11 (the lowest level of support) to 55 (the highest level of perceived social support). The DUFSS presented adequate psychometric properties, showing a Cronbach’s alpha value of 0.9 and strong construct validity [21,22]. We included the DUFSS in our study to corroborate previous findings [23,24,25,26,27] showing a close relationship between living with HF and social support. Thus, we analysed convergent validity between the LW-CI-HF scale and the DUFSS.

#### 2.4.4. Modified Version of the Satisfaction with Life Scale (SLS-6)

The SLS-6 was used to measure satisfaction with life in the patient living with HF [28,29]. The SLS-6 is a 6-item scale in regard to 5 areas: physical (1), psychological wellbeing (2), social relations (3), leisure (4) and financial situation (5). Each item is scored on a Likert scale from 0 (totally unsatisfied with life) to 10 (totally satisfied with life). The modified version of the SLS was used because the original version of the scale (with 7 items) contains an item related to student life satisfaction, which is not pertinent for the target population. The SLS-6 presented satisfactory psychometric properties, with a Cronbach’s alpha of 0.8 and internal validity values ranging from 0.4 to 0.7 [28,29]. This scale was included in our study to analyse convergent validity and verify the results of previous studies in HF [12,24,25,27], where satisfaction with life had a close relationship with the patient’s daily living.

#### 2.4.5. Brief Version of the WHO Quality of Life Scale (WHOQOL-BREF)

The WHOQOL-BREF [30,31] is a short measuring scale to evaluate the overall quality of life of a person. This instrument is a self-reported scale that contains 24 items grouped into the following 4 domains: (1) physical health, (2) psychological relationships, (3) social relationships and (4) environment. Each item is scored from very dissatisfied (1) to very satisfied/very good quality of life (5), and the total score for each domain ranges from 4 to 20. The patient with a higher score has a better quality of life [31]. The Spanish version of the scale had satisfactory psychometric properties in an older Spanish-speaking population, with a Cronbach’s alpha of 0.90 [30]. We included the WHOQOL-BREF to analyse the convergent validity of the LW-CI-HF scale, as a correlation between living with a chronic disease such as HF and the quality of life was identified in previous studies [11,12,23,25,26,27].

#### 2.4.6. Patient-Based Global Impression of Severity Scale (PGIS)

The PGIS is a global index that may be used to assess self-perception of disease severity [32]. The PGIS is a scale of symptom severity that is intuitively understandable to healthcare professionals [32]. This scale is rated on a 6-point Likert scale, using a range of responses from 0 (not ill at all) to 5 (extremely ill). The PGIS has excellent construct validity and has been widely used in studies of chronic diseases [33].

### 2.5. Data Collection

Data collection was carried out between May 2018 and June 2019 in the primary and secondary healthcare centres in Spain and Colombia. The principal researcher of the study (L.A) ensured that all researchers involved in this process understood the established steps before starting the data collection procedure. Those rating this process were healthcare professionals (general or cardiovascular specialist nurses or physicians). For homogeneity purposes, the principal investigator developed a standardized protocol of procedures for all the centres from Spain and Colombia. The main steps were as follows: participants who fulfilled the established criteria were approached through the healthcare professionals during routine medical visits, and an invitation letter and the participant information sheet were provided to explain this study. Patients were invited for a second visit with the healthcare professional in his/her corresponding healthcare centre to complete all the questionnaires, which were self-reported and took an average of 30–40 min per patient.

The data collection procedure was also designed to minimize possible mistakes. For the retest, patients completed the LW-CI-HF scale a second time at home. The instructions for the retest were explained to the patient after completing all questionnaires during the first visit. The LW-CI-HF scale was enclosed in a stamped envelope with the principal investigators postal address to be sent in an easy and cost-free way for the patient. According to international experts in validation studies [34], a minimum sample of 50 subjects with a response time of 7 to 10 days was required for the retest.

### 2.6. Data Analysis

Sociodemographic characteristics and historical data of the HF were presented with descriptive statistics (central tendency measures, proportions) with normal distribution, and nonparametric tests were used. Additionally, the following psychometric attributes of the LW-CI-HF scale were tested.

Feasibility and acceptability were tested. The quality of the data was considered satisfactory if 95% of the data were computable. The limit for missing data was <5% [35]. Floor and ceiling effects were deemed acceptable if they were <15%, and the skewness was expected to be between −1 and +1 [36].

Cronbach’s alpha coefficient with a criterion value ≥0.7 was tested for internal consistency [37], item-total correlation (corrected for overlap; criterion value, r_s_ ≥ 0.3) [38], inter-item correlation (criterion value, 0.2 ≤ r_s_ ≤ 0.7) [39] and item homogeneity (criterion value > 0.3) [40].

The test–retest reliability required 105 patients with HF, using the weighted kappa (with quadratic weights) for items (standard: >0.4 moderate) [41] and the intraclass correlation coefficient (one-way, random effect) for domains and total score. Values ≥ 0.6 were considered acceptable [42].

Precision, or the ability of the scale to detect small differences, for each LW-CI-HF scale domain and for the total scale was estimated by means of the standard error of measurement (SEM), according to the formula:

(1) $$SEM=SD_{pooled}*\sqrt{(1-r_{xx}})$$

where pooled standard deviation (SD)*_pooled_* =

(2) $$SD_{pooled\;}=\sqrt{\left(SD_{1}^{2}+\;SD_{2}^{2}\right)/2}$$

and r_xx_ is the HF of the test–retest. A SEM value < ½ SD was used as the criterion of acceptable precision [43,44].

Construct validity. For convergent validity, a moderate (0.3 ≤ r_s_ ≤ 0.5) or strong relationship (r_s_ > 0.5) [45,46,47] was hypothesized between the LW-CI-HF scale and DUFSS, WHOQOL-BREF and SLS-6, according to evidence in long term conditions and in particular, HF [11,12,23,24,25,26,27]. Spearman’s rank correlation coefficients were calculated for this purpose. Internal validity, defined as the intercorrelations between the LW-CI-HF scale dimensions (standard, r_s_ = 0.3−0.7) [36,38] was also tested. Known-group validity of the LW-CI-HF scale was analysed in the sample grouped by sociodemographic data, functional classification according to the NYHA classification and PGIS scores [48,49,50]. Hypotheses related to significant differences in LW-CI-HF scores by gender, marital status, employment situation, NYHA classification and PGIS levels were set, following the literature [49,51]. We chose the variables for grouping the patients based on our previous research on the construct “living with chronic disease” and on the knowledge about the determinants of clinical outcomes in patients with HF [49]. For group comparison, we used the Mann–Whitney U and Kruskal–Wallis statistics tests with Bonferroni post hoc test correction.

### 2.7. Ethical Aspects

The study was approved by the research ethics committee of the centre of the principal investigator (reference number: 2017.099) and all included centres from Spain and Colombia. This validation study conforms was adjusted to the principles outlined in the Declaration of Helsinki (1964) of Law 14/2007 on Biomedical Research and Law 15/1999 on the Protection of Personal Data. All participants signed their informed consent after receiving pertinent oral and written information and before inclusion in the study. Patients participated voluntarily without any economical compensation. The principal investigator of the study (L.A.) was responsible for guaranteeing the confidentiality of participants’ identity. Following the rules of the ethics committee, all questionnaires were kept locked in the office of the principal investigator.

## 3. Results

Of the 640 patients invited to participate in the research, 603 composed the sample: 321 from Colombia and 282 from Spain. The main sociodemographic characteristics of the sample were as follows: 53.1% were men, the mean age was 71.7 years old (standard deviation, SD: 11.4; range: 23–96), 59.4% were married, 49.80% were retired and 65.7% had a basic or primary education level. Patients were diagnosed with HF at 63.9 years old (SD: 12.0; range: 21–91) on average, and the average time since diagnosis was 7.8 years (SD: 7.4; range: 0.1–55). Nearly half (49.3%) were in NYHA class II. A detailed description of the sociodemographic characteristics of the sample and the historical data of HF are shown in Table 1.

### 3.1. Feasibility and Acceptability

Regarding the feasibility and acceptability of the LW-CI-HF scale, there were no missing data, except for item 15 (Domain 3—Self-management). All acceptability parameters fulfilled the standard criteria. The LW-CI-HF total scale results showed no floor or ceiling effects (0.2 and 4.2%, respectively), and all items reached the maximum score range. Skewness values were between −0.2 and −0.8. For further information, see Table 2.

### 3.2. Reliability

In the analysis of the internal consistency, Cronbach’s alpha was >0.7 for all domains, ranging from 0.8 to 0.9. For the total score, Cronbach’s alpha was 0.9. Item-total corrected correlation values were higher than 0.3 in all items, except for Domain 2—Coping, where lower values were identified (0.2). Item homogeneity index values ranged from 0.4 (Domain 2—Coping) to 0.6 (Domain 1—Acceptance) (see Table 2).

The test–retest reliability of the LW-CI-HF scale was determined with 105 patients with HF, 55 from Spain and 50 from Colombia, 58.1% men and a mean age of 70.91 (SD: 12.43; range: 35–96) years (see Table S1). The ICC was 0.9 on the total scale and ranged between 0.7 and 0.8 for the dimensions. The weighted kappa index ranged from 0.6 (items 18 and 25) to 0.8 (item 9). See Table 2 and Supplementary Material (Tables S1 and S2) for further information on the retest sample characteristics and results.

### 3.3. Precision

The SEM for the LW-CI-HF scale total score was 6.5 (<½ SD), and for the dimensions ranged from 1.48 (Domain 3—Self-management) to 2.82 (Domain 2—Coping). In Domains 2—Coping, 3—Self-management and 4—Integration of the scale, the value of SEM was <½ SD (see Table 2).

### 3.4. Validity

Regarding content validity, patients referred to the LW-CI-HF scale as an easy, clear and short questionnaire. Only some of the patients (2%) mentioned that items related to Domain 4—Integration were not applicable for people living with a long-term condition such as HF. Table 3 shows the values related to convergent validity. Strong correlation coefficients between the LW-CI-HF scale total and SLS-6 and DUFSS were found (0.7). The LW-CI-HF scale also showed strong correlations with all domains of the WHOQOL-BREF except for Physical Health in Domain 1 (0.3).

All domains of the LW-CI-HF scale showed correlation values greater than 0.7, except Domain 1—Acceptance, which showed poorer correlations with the other dimensions. Therefore, there is a high internal consistency in the scale. More details are shown in Table 3.

The total score of known-group validity analysis showed statistically significant differences for marital status, employment situation, HF classification and patient self-perception of severity. See Table 4 for additional information.

## 4. Discussion

The results of this validation study suggest that the LW-CI-HF scale is a reliable and valid instrument for the assessment of how patients with HF are living with the disease. The wide sample included in this study was a heterogeneous representation of people living with a chronic condition and irreversible condition such as HF [10,12]. Overall, the psychometric properties of the scale were generally satisfactory. Due to the investigators’ close supervision during the data collection, the quality of the data was good, and feasibility and acceptability attributes were adequate. These findings indicate that the LW-CI-HF scale has an adequate distribution of the scores suitable for the HF population.

The reliability of the LW-CI-HF scale was also satisfactory. Precision was adequate for three of the subscales and for the total scale score, suggesting the suitability of the LW-CI-HF scale total score for assessing change after treatment or over time [52].

Regarding convergent validity, as we hypothesized, the process of living with HF presents a strong relationship with social support and satisfaction with life [12,23,24,25,26,27]. In this study, we used the measures DUFSS and SLS-6 to evaluate those concepts. According to the authors in the HF field [23,24,53], social support refers to health professionals’ support, family and friends’ help and/or animals’ company. The spouse and children were identified as the key supporters in patients living with HF [25,27]. More concretely, researchers in HF identified the following benefits that social support generates in the patient when living with HF: feeling less stressed and more comfortable in their daily living with the adversities that the HF generates [23], better acceptance of and coping with the diagnosis and the progression of the disease [23,24], the reinforcement of self-care strategies for enhancing positive daily living with the disease [23,26] and promoting better psychological adjustment and management of the symptoms [23]. In this regard, having poor social support influences patients in a negative way, leading to feelings of insecurity, lower self-esteem and negative daily living with HF [27,54]. Therefore, according to the results that emerged in this validation study and other researchers’ findings in this field, we could suggest that social support influences daily living with HF. Nevertheless, to confirm these results, other analyses, such as multiple linear regression models, are recommended in future studies.

Regarding convergent validity results, we have also identified a strong relationship between living with HF and satisfaction with life, supporting one of the hypotheses established for this validation study. We confirmed the close relationship between living with HF and satisfaction with life that different researchers mention in their studies [12,53]. Thus, better living with HF is related to a more satisfactory life because HF influences several spheres of the patient, such as psychological wellbeing, social relationships and overall daily living satisfaction. In addition, according to the results that emerged in the convergent validity, this study highlights the moderately strong relationship between living with HF and quality of life. In our research, psychological health, social relationships or environmental domains have a strong relationship with daily living with HF, whereas physical health presents a slight relationship with living with HF. This result is reinforced with those of other studies in this field [23,25,26,27] showing that quality of life is more related to daily living aspects such as emotional, social or spiritual aspects than to physical health [12,27,53]. However, these results should be taken with caution because a causal relationship cannot be attributed due to the design of this study. Therefore, we advocate for the development of future longitudinal studies with other types of analysis, as multiple linear regression analysis is used to verify these results.

Regarding internal validity, our findings showed that all domains are intercorrelated and measure the same construct: living with HF. However, Domain 1—Acceptance is always the first attribute to achieve positive daily living with the disease [11]. We conclude that only when the person has accepted his/her illness, and thus the new situation, can he/she move on to another attribute of living with chronic illness. In this sense, other authors in the chronic condition field [9,15,20] supported this result, showing that acceptance could be an internal process different from the illness through the patient recognizing and assuming reality. However, we will verify this aspect in future studies testing the different domains of the LW-CI-HF scale using Rasch analysis.

Finally, the results regarding known-groups validity showed that the LW-CI-HF scale discriminates depending on marital status, employment situation or the severity of HF according to the NYHA classification and PGIS [48,49,50]. We know that HF produces a considerable disruption in patients’ marital and working lives, and these variables are important determinants of clinical outcomes in HF [48,50]. Regarding the severity of the disease, in previous studies carried out in other long-term conditions, such as Parkinson’s disease [15], we found that patients in early stages of the disease present a better degree of living with Parkinson’s disease (positive living) than patients in more advanced stages (negative living), and we hypothesized that similar results could be found in patients with HF. Other researchers [6,55] also showed that people living with HF may have more sudden changes in symptoms in a short time related to the severity of their structural and/or functional cardiac abnormalities than people with other chronic diseases such as cancer, chronic obstructive pulmonary disease or diabetes mellitus [6,8,56].

Overall, the psychometric data of this validation study suggest that the LW-CI-HF scale is a reliable and valid instrument to measure the complex process of living with HF in a Spanish-speaking population. In this way, implications of this study are related with the usefulness of the LW-CI-HF scale in clinical practice and research. The LW-CI-HF scale is the first holistic instrument available to evaluate how the patient lives with the illness in clinical practice and research. If we are to move to a model of personalized and holistic care, health and social care professionals need to use reliable and valid measuring scales such as LW-CI-HF scale in current clinical practice to evaluate how the patient is living with one or more than one chronic diseases and consequently, develop individualized and patient-centred care plans to prevent typical psychosocial issues or disorders while living with HF and/or reinforce those positive aspects of the person to empower and achieve positive daily living with HF. Although results emerged in this study should be taken with caution, the LW-CI-HF scale could be used by cardiovascular healthcare practitioners as a complement to conventional generic health-related quality of life measures, as a basis for evaluation where interventions may affect both health and social care outcomes, and in comparing outcomes and resource allocation across different HF issues such as denial, depression, dissatisfaction or poor quality of life [12,25,27,57,58]. Thus, we advocate the necessity to incorporate reliable and valid measuring scales, such as the LW-CI-HF scale, in clinical practice to personalize the patient’s experience with the disease and tackle the person as a unique individual. Using the LW-CI-HF scale in routine clinical practice could result in optimal healthcare utilization without sacrificing quality of life and economic costs because it could ensure more effective risk stratification and the early identification of people with higher needs for more complex care and at risk of poor self-management. Another implication of this study is related with health promotion because the LW-CI-HF scale could facilitate the implementation of educational interventions for people living with HF, increasing their efficiency in the collection of information and integration of services. Finally, implications of this study are also relevant for the quality requirements of healthcare system strategies regarding chronic conditions, especially in regard to the assessment of personal care and support in an attempt to tackle physical, emotional, spiritual and social difficulties.

The findings presented here should be viewed in light of several limitations. Firstly, the results could be influenced by the presence of the researcher with the patient during the data collection. However, none of the patients included in the study expressed feeling uncomfortable or influenced by the researcher. Besides, it is possible that the participation of two Spanish-speaking countries in the study could be seen as a limitation related to external validity. In this way, we propose further validation studies in other Spanish-speaking countries as well as other languages. Finally, in addition to HF, patients could have other chronic conditions that were not taken into account in this study. The aim of our study was to validate the LW-CI scale in a sample of patients with HF because this is a chronic condition that supposes a burden for patients and families per se. Further studies are needed to assess the construct living with a chronic illness in patients with co-morbidities. Nevertheless, this study also presents several strengths such as the heterogeneity of the sample included from two different countries, which supports greater consistency of the results at least for this cultural and linguistic setting. Another strength is that the sample could be considered representative of the HF population for the heterogeneity in the severity of the symptoms according to the diagnostic criteria of the illness. Finally, this scale could be used in other Spanish-speaking countries.

## 5. Conclusions

According to this first validation study, we can conclude that the LW-CI-HF scale is a feasible, reliable and valid measure to evaluate the process of living with HF in Spanish-speaking population. Nowadays, this is the only available instrument in clinical practice and research to evaluate how a person with HF is living with the disease in a comprehensive way from the patient’s perspective. However, results should be taken with caution when using it in clinical practice, so further studies are proposed in the area.

## Figures and Tables

**Table 1:** Sociodemographic characteristics of the patients (*n* = 603) and historical data of the disease.

Demographical Variables	Response Options	Total Patients Living with HF *N* (%)
**Gender**	Male Female	320 (53.1) 283 (46.9)
**Marital status**	Married	358 (59.4)
	Single	50 (8.3)
	Widower	145 (24)
	Others	50 (8.3)
**Employment situation**	Active working	57 (9.5)
	House keeper	199 (33)
	Retired	300 (49.8)
	Others	47 (7.8)
**Educational level**	Primary studies	396 (65.7)
	Secondary studies	143 (23.7)
	University studies	52 (8.6)
	Others	12 (2)
**Treatment for HF**	Yes	236 (39.1)
	No	367 (60.9)
**New York Heart Association classification**	Class I	163 (27)
	Class II	297 (49.3)
	Class III	113 (18.7)
	Class IV	29 (4.8)
**PGIS**	0 (Normal)	80 (13.3)
	1–2 (Minimal/Mild)	146 (24.2)
	3 (Moderate)	260 (43.1)
	4–5 (Severe/very severe)	117 (19)
	**Range**	**Mean (Standard Deviation)**
**Age**	23–96 years	71.7 (11.4) years
**Age at diagnosis**	21–91 years	63.9 (12) years
**Duration with HF**	1 month–55 years	7.8 (7.4) years

HF: heart failure; PGIS: Patient-Based Global Impression of Severity Scale.

**Table 2:** Feasibility/acceptability, reliability and precision of the LW-CI-HF scale.

	LW-CI-HF Scale					
	Domain 1— Acceptance	Domain 2—Coping	Domain 3—Self-Management	Domain 4—Integration	Domain 5—Adjustment	Total Score
**Data Quality (% fully computable data)**	100	100	99.5	100	100	99.5
**Mean (SD)**	11.1 (4.2)	19.6 (6.2)	11.1 (3.8)	15.4 (4.1)	15.7 (6.5)	72.9 (20.1)
**Floor Effect (%)**	2.2	0.3	0.3	0.3	0.7	0.2
**Ceiling Effect (%)**	24.4	10.8	19.8	24.7	22.7	4.2
**Skewness**	−0.6	−0.5	−0.4	−0.8	−0.2	−0.3
**Cronbach’s Alpha**	0.9	0.8	0.8	0.8	0.9	0.9
**Item-Total Correlation**	0.48–0.74	0.27–0.59	0.37–0.57	0.36–0.65	0.44–0.76	-
**Item Homogeneity**	0.6	0.4	0.5	0.5	0.6	-
**Reproducibility (ICC)**	0.7	0.8	0.8	0.8	0.7	0.9
**Precision (SEM)**	1.9	2.8	1.5	1.5	2.7	6.5

LW-CI-HF scale: Living with Chronic Illness—Heart Failure Scale; SD: standard deviation; SEM: standard error of measurement; ICC: intraclass correlation coefficient.

**Table 3:** Convergent validity and internal validity of LW-CI-HF scale.

		LW-CI-HF Scale					
		Domain 1— Acceptance	Domain 2— Coping	Domain 3— Self-Management	Domain 4—Integration	Domain 5—Adjustment	Total Score
**Convergent validity**	**DUFSS**	0.3	0.6	0.6	0.6	0.6	0.7
	**SLS-6**	0.4	0.6	0.5	0.6	0.6	0.7
	Satisfaction—Physical	0.4	0.4	0.4	0.5	0.4	0.5
	Satisfaction—Psychological well-being	0.4	0.5	0.5	0.6	0.6	0.7
	Satisfaction—Social relations	0.3	0.5	0.5	0.6	0.5	0.6
	Satisfaction—Leisure	0.3	0.5	0.4	0.6	0.5	0.6
	Satisfaction—Financial situation	0.3	0.3	0.3	0.4	0.4	0.4
	**WHOQOL-BREF**	0.3	0.6	0.6	0.6	0.6	0.7
	WHOQOL-BREF—Physical Health	0.4	0.2	0.2	0.3	0.4	0.3
	WHOQOL-BREF—Psychological Health	0.5	0.5	0.5	0.6	0.6	0.7
	WHOQOL-BREF—Social relationships	0.3	0.4	0.5	0.5	0.5	0.5
	WHOQOL-BREF—Environmental	0.4	0.5	0.5	0.6	0.5	0.6
**Internal validity**	**Domain 2—Coping**	0.3	-	-	-	-	-
	**Domain 3—Self-management**	0.2	0.7	-	-	-	-
	**Domain 4—Integration**	0.4	0.8	0.7	-	-	-
	**Domain 5—Adjustment**	0.3	0.7	0.8	0.7	-	-

DUFSS: Duke–UNC Functional Social Support Questionnaire; LW-CI-CHF scale: Living with Chronic Illness—Heart Failure Scale; PGIS: Patient-Based Global Impression of Severity Scale; WHOQOL-BREF: Brief Version of the World Health Organization Quality of Life Instrument. All correlation coefficients, *p* < 0.01.

**Table 4:** Known-groups validity.

Variable	Categories	LW-CI-HF Scale	*p*
**Gender**	Men	72.0 ± 19.6	0.21
	Women	73.9 ± 20.6	
**Marital status**	Single	71.2 ± 18.7	0.04
	Married	74.8 ± 19.5	
	Widower	68.6 ± 22.2	
	Other	73.5 ± 17.4	
**Employment situation**	Employee	75.2 ± 18.3	0.01
	Housewife	79.2 ± 20.0	
	Retired	68.8 ± 19.1	
	Other	69.2 ± 21.8	
**New York Heart Association Classification**	Class I	80.8 ± 20.1	<0.001
	Class II	73.2 ± 17.9	
	Class III	64.3 ± 20.2	
	Class IV	57.7 ± 21.3	
**PGIS**	0 = Normal	77.3 ± 17.4	<0.001 *
	1–2 = Minimal/Mild	76 ± 17.4	
	3 = Moderate	76.1 ± 19.7	
	4–5 = Severe/Very severe	58.78 ± 19.7	

LW-CI-HF Scale: Living with Chronic Illness—Heart Failure Scale; PGIS: Patient-Based Global Impression of Severity Scale. * Significant only for severe/very severe level vs. the rest of levels. Mann–Whitney test for gender, Kruskal–Wallis test for the rest of variables, with Bonferroni correction. Mean ± standard deviation.

## Data Availability

The data presented in this study are available on request from the corresponding author. The data are not publicly available due to privacy and ethical aspects.

## Supplementary Materials

The following are available online at https://www.mdpi.com/1660-4601/18/2/572/s1, Table S1: Characteristics of the retest sample, Table S2: Test–retest reliability.
