# Supplementary material for: Psychometric Validation of the Living with Chronic Illness Scale in Patients with Chronic Heart Failure
Source: Int J Environ Res Public Health. 2021 Jan 12;18(2):572. doi: 10.3390/ijerph18020572 (PMC7828024; doi:10.3390/ijerph18020572)
Supplement: Supplementary file 1 [file ijerph-18-00572-s001.pdf]

SUPPLEMENTARY MATERIAL 1. Characteristics of the retest sample

Demographical variables	Options	Total patients living with CHF	
		N	%
**Country**	Spain	55	52.4
	Colombia	50	47.6
**Gender**	Men	61	58.1
	Women	44	41.9
**Educative level**	Primary	62	59.0
	Secondary	26	24.8
	University	15	14.3
	Other	2	1.9
**Employment**	Active worker	12	11.4
	House keeper	17	16.2
	Retired	62	59.0
	Others	14	13.3
**New York Heart Association classification**	Class I	46	43.8
	Class II	42	40.0
	Class III	10	9.5
	Class IV	7	6.7
		**Mean (Standard Deviation)**	**Range**
**Age**		70.9 (12.4) years	35 - 96 years
**Duration of treatment**		4.6 (8.3) years	0 - 55 years

Table 2. Test-retest reliability

	Test			Retest				%	Weighted	
	N	Mean	SD	N	Mean	SD	p^*	agreement	kappa	ICC
**Domain 1-Acceptance**	**105**	**12.5**	**3.6**	**105**	**11.9**	**3.7**	**0.1**			**0.72**
Item 1	105	3.4	1.1	105	2.9	1.1	0.6	95.1	0.7	
Item 2	105	3.6	0.8	105	3.5	0.9	0.1	96.5	0.6	
Item 3	105	3.1	1.2	105	2.9	1.2	0.1	93.5	0.6	
Item 4	105	2.8	1.3	105	2.6	1.2	0.1	93.7	0.7	
**Domain 2-Coping**	**105**	**16.8**	**6.5**	**105**	**16.7**	**5.9**	**0.3**			**0.8**
Item 5	105	2.3	1.4	105	2.5	1.3	0.2	92.7	0.7	
Item 6	105	2.4	1.4	105	2.2	1.3	0.2	92.0	0.7	
Item 7	105	2.1	1.4	105	1.9	1.4	0.1	92.9	0.7	
Item 8	105	3.1	1.1	105	3.1	1.1	0.4	96.5	0.7	
Item 9	105	1.8	1.5	105	1.9	1.4	0.5	94.5	0.8	
Item 10	105	2.9	1.1	105	2.9	1.1	0.7	95.9	0.7	
Item 11	105	2.2	1.2	105	2.2	1.2	0.4	92.7	0.6	
**Domain 3-Self-management**	**105**	**9.9**	**3.3**	**105**	**9.9**	**3.2**	**0.8**			**0.8**
Item 12	105	1.6	1.5	105	1.7	1.4	0.6	94.3	0.8	
Item 13	105	2.6	1.1	105	2.5	1.0	0.3	95.5	0.7	
Item 14	105	2.9	0.9	105	2.9	0.9	0.4	96.4	0.7	
Item 15	105	2.8	1.2	105	2.8	1.1	0.8	94.2	0.7	
**Domain 4-Integration**	**105**	**14.3**	**3.7**	**105**	**14.1**	**3.6**	**0.1**			**0.8**
Item 16	105	2.9	1.1	105	2.9	0.9	0.7	96.2	0.7	
Item 17	105	3.1	0.9	105	3.1	0.9	0.8	97.3	0.7	
Item 18	105	2.8	1.1	105	2.8	1.1	0.8	93.9	0.6	
Item 19	105	2.6	1.9	105	2.5	1.1	0.2	94.8	0.6	
Item 20	105	2.9	0.9	105	2.8	0.9	0.4	96.9	0.7	
**Domain 5-Adjustment**	**105**	**13.3**	**5.3**	**105**	**13.6**	**5.8**	**0.4**			**0.7**
Item 21	105	2.5	1.3	105	2.5	1.3	0.8	93.5	0.7	
Item 22	105	1.7	1.3	105	1.8	1.4	0.7	91.0	0.6	
Item 23	105	2.4	1.2	105	2.5	1.2	0.1	94.3	0.7	
Item 24	105	2.9	1.1	105	2.9	0.9	0.9	96.8	0.7	
Item 25	105	2.1	1.3	105	2.1	1.4	0.7	89.8	0.6	
Item 26	105	1.7	1.3	105	1.9	1.3	0.2	92.2	0.6	
**Total score**	**105**	**66.9**	**17.3**	**105**	**66.2**	**17.4**	**0.1**			**0.9**

SD = Standard deviation; ICC = Intraclass correlation coefficient; * Wilcoxon test.
